# Uncovering Non-Invasive Biomarkers in Paediatric Severe Acute Asthma Using Targeted Exhaled Breath Analysis

**DOI:** 10.3390/metabo15040247

**Published:** 2025-04-03

**Authors:** Sarah van den Berg, Annabel S. Zaat, Isabel F. van der Poel, Yoni E. van Dijk, Simone Hashimoto, Niels W. P. Rutjes, Suzanne W. J. Terheggen-Largo, Bart E. van Ewijk, Claudia Gagliani, Fleur L. Sondaal, Job B. M. van Woensel, Anke-Hilse Maitland-van der Zee, Paul Brinkman, Susanne J. H. Vijverberg, Berber Kapitein

**Affiliations:** 1 Paediatric Intensive Care Unit, Emma Children’s Hospital, Amsterdam University Medical Centre, Location AMC, 1105 AZ Amsterdam, The Netherlands; sarah.vandenberg@amsterdamumc.nl (S.v.d.B.); j.b.vanwoensel@amsterdamumc.nl (J.B.M.v.W.); 2 Department of Pulmonary Medicine, Amsterdam University Medical Centre, Location AMC, 1105 AZ Amsterdam, The Netherlands; y.e.vandijk@amsterdamumc.nl (Y.E.v.D.); s.hashimoto@amsterdamumc.nl (S.H.); a.h.maitland@amsterdamumc.nl (A.-H.M.-v.d.Z.); p.brinkman@amsterdamumc.nl (P.B.); s.j.vijverberg@amsterdamumc.nl (S.J.H.V.); 3 Faculty of Medicine, University of Amsterdam, 1105 AZ Amsterdam, The Netherlands; a.s.zaat@hotmail.com (A.S.Z.); i.f.vanderpoel@amsterdamumc.nl (I.F.v.d.P.); f.l.sondaal@amdserdamumc.nl (F.L.S.); 4 Department of Paediatric Pulmonology, Emma Children’s Hospital, Amsterdam University Medical Centre, Location AMC, 1105 AZ Amsterdam, The Netherlands; n.w.rutjes@amsterdamumc.nl (N.W.P.R.); s.w.terheggenlagro@amsterdamumc.nl (S.W.J.T.-L.); 5 Department of Paediatric Medicine, Tergooi Medical Centre, 1201 DA Hilversum, The Netherlands; bvanewijk@tergooi.nl; 6 PROMISE Department, Division of Respiratory Medicine, University of Palermo, 90127 Palermo, Italy; dott.claudiagagliani@gmail.com

**Keywords:** severe acute asthma, paediatrics, paediatric intensive care unit, breathomics, volatile organic compounds, thermal desorption gas chromatography–mass spectrometry

## Abstract

Background: Severe acute asthma (SAA) in children can be life-threatening. There has been a significant rise in paediatric intensive care unit (PICU) admissions due to SAA over the past two decades. While asthma is a heterogeneous disease, its underlying pathophysiological pathways remain underexplored. This study aimed to assess the value of non-invasive targeted exhaled breath metabolomics analysis to better characterise SAA. Methods: Breath samples from 17 children admitted to the PICU with SAA (cases) and 27 children with controlled severe asthma (controls) were analysed using thermal desorption gas chromatography–mass spectrometry (TD-GC-MS). Results: A targeted volatile organic compound (VOC) analysis identified 25 compounds, of which 16 were shared between groups. Four VOCs were significantly more often present in SAA, and nine VOCs exhibited higher concentrations in SAA. Longitudinal analysis of VOCs from follow-up samples of 10 cases showed no significant temporal differences, reinforcing the reproducibility of identified biomarkers. Conclusions: This study exemplifies the potential of exhaled breath analysis to provide insights into the molecular background of SAA. Breath metabolomics may enable early recognition of severe asthma attacks and preventive therapeutic interventions in children with severe asthma.

## 1. Introduction

Asthma is a chronic pulmonary disease that consists of bronchial hypersensitivity in reaction to triggers such as viral upper-airway infections or environmental factors like dust or mould. It may subsequently lead to bronchospasms and wheezing. The overall prevalence of childhood asthma worldwide is 10.2% [1]. While children with an asthma exacerbation generally respond well to therapy with bronchodilators and oral corticosteroids, children with severe acute asthma (SAA) have a severe asthma exacerbation that is unresponsive to this standard therapy, and these children have to be admitted to a paediatric intensive care unit (PICU). Over the past twenty years, admissions for SAA have shown a three- to fourfold increase worldwide [2,3,4]. The standard therapy for SAA consists of intravenous bronchodilators (salbutamol or theophylline) and systemic corticosteroids. It is important to recognise symptoms of SAA at an early stage and start prompt treatment, but this is difficult, as its onset varies widely and pre-stage symptoms can be mild [5]. SAA has a high morbidity and may lead to fatalities; moreover, it has been shown to have a significant mental burden on both the patient and its caregivers [6]. The observed increase in cases of SAA at the PICU is not preceded by an increase in chronic asthma cases [7]; thus, it may be suggested that this increase is explained by a shift in the endotype of (severe) asthma.

Asthma is a heterogeneous disease with several different clinical characteristics and underlying pathophysiologic mechanisms [8]. The ‘phenotype’ includes demographic and clinical aspects such as atopy, symptoms, triggers, and body weight, and it shows a large variety. The ‘endotype’ consists of various underlying pathobiological pathways on a cellular and molecular level that partly explain differential responses to pharmacological interventions [9]. Asthma used to be regarded as a type 2 T-helper-mediated inflammatory disease, which is eosinophil-driven and steroid sensitive. In addition, other pathobiological mechanisms exist, such as non-type 2 asthma, e.g., neutrophil-driven asthma, which is less responsive to steroids. Even more underlying pathobiological mechanisms may exist [10].

The discovery of these underlying mechanisms in children with asthma has been difficult because most techniques are invasive [10]. Breathomics, the metabolic study of exhaled air, is a minimally invasive bioanalytical technique analysing exhaled breath on various compounds, forming a so-called ‘breath print’ [11]. This breath print is unique for every human and contains hundreds of volatile organic compounds (VOCs), which are often products of (patho)physiological processes. There are several different types of VOCs, including endogenous VOCs, which reflect either metabolic pathways taking place in the local environment of the lungs or systemic metabolic processes associated with, for instance, inflammatory or oxidative activity, or exogenous VOCs, derived from the environment [12,13]. VOCs can be measured by breathomics and analysed using gas chromatography and mass spectrometry (GC-MS) [14]. The utility of VOCs has been proven in several diseases such as COPD, cystic fibrosis, lung cancer, and non-pulmonary diseases such as colorectal or breast cancer [15]. In asthma, certain VOCs have been identified that are specific for the disease and may be influenced by the disease activity [16]. For example, inflammatory cells active during an asthma exacerbation produce reactive oxygen species (ROS), causing oxidative stress. These ROS promote the degradation of polyunsaturated fatty acids present in structures such as the bronchial epithelium, and various volatile hydrocarbons are formed and can be measured [16]. Moreover, it has been found that the composition of exhaled VOCs changed two months before clinical symptoms of an asthma exacerbation appeared and could predict the exacerbation risk with a sensitivity of 79–88% and specificity of 75–100% [17,18]. Finally, certain VOCs were able to discriminate between controlled and uncontrolled severe asthma, ensuring timely intervention and management [19].

These studies show the potential of VOCs in childhood asthma to illustrate disease activity, predict future exacerbations, and enable control classification, thereby supporting clinical decisions. All of the current studies are being conducted in children with moderate to severe asthma, neglecting the patient group requiring a PICU admission. Breathomics may have the potential to recognise SAA at an early stage before patients and physicians can. Additionally, breath-based metabolomic profiling may help elucidate underlying pathobiological mechanisms, enabling a more targeted treatment regimen instead of the current one-size-fits-all approach. The validation of previous results in a standardised manner is an essential step towards the possibility of using breath analysis in clinical care [17,18,19]. To our knowledge, the mentioned VOCs related to asthma exacerbations have not yet been validated in children with SAA requiring PICU admission, so the potential of these specific VOCs in this patient group is unknown. Therefore, in this study, our first aim was to conduct a literature review of VOCs measured during an asthma exacerbation; then, our second aim was to use these identified VOCs to perform a targeted exhaled breath analysis to try and differentiate breath prints between SAA and controlled asthma.

## 2. Materials and Methods

### 2.1. Systematic Literature Review to Select VOCs

A broad systematic search of the PubMed Central library was performed by A.Z. in December 2022 to identify VOCs previously associated with asthma exacerbations, using a similar approach to that of Kos et al. [20]. The following search terms were used: asthma [Mesh] AND (GC-MS OR GCMS OR volatile organic compound * OR VOC OR exhaled breath) AND (exacerbation OR acute * OR severe *), without the use of additional filters. Selection for full-text examination was based on the title and/or abstract. Inclusion criteria were articles that used the same method for GC-MS and breath measurements taken during an asthma exacerbation. In addition, the preliminary report of Mager et al. was used to exclude contamination of exogenous VOCs originating from the same sampling set-up that was used [21].

### 2.2. Clinical Study Design

A case-control study was conducted using data from the MACU-PICU study (Multisystem phenotyping in severe acute asthma at the PICU, NL70251.018.19) and the PANDA study (Paediatric Asthma Non-Invasive Diagnostic Approaches, NL67105.018.18). MACU-PICU is a single-centre prospective observational cohort of children admitted to the PICU of the Emma Children’s Hospital at the Amsterdam University Medical Centre (AUMC), the Netherlands. PANDA is a multi-centre prospective observational cohort study of Dutch children with severe asthma recruited at the outpatient asthma clinics at the Amsterdam University Medical Centre and Tergooi Medical Centre, the Netherlands [22]. Ethical approval was provided by the ethical review committee of AUMC. Furthermore, all study procedures were performed according to the relevant guidelines and regulations in the Declaration of Helsinki.

### 2.3. Study Population

Cases. Children > two years of age admitted to the PICU with SAA receiving salbutamol or theophylline intravenously (IV) from the MACU-PICU study were included as cases (*n* = 17). Due to the heterogeneous clinical symptoms, SAA was only diagnosed if patients met the following three criteria: (a) diagnosed as having SAA by a paediatric intensivist according to the criteria of the Dutch protocol for acute asthma; (b) a confirmation of the diagnosis by a paediatric pulmonologist; and (c) a positive effect of salbutamol IV or theophylline IV with a decrease in the Qureshi asthma score [23]. For the breath sampling, patients had to be stable enough, according to the attending physician. Children were excluded if other significant respiratory comorbidities (e.g., cystic fibrosis, primary ciliary dyskinesia, congenital lung disorders) or severe immune disorders existed. Furthermore, patients or custodial caregivers had to have a sufficient understanding of the Dutch language to understand and sign the informed consent form. Breath samples were collected during admission and after approximately 6 weeks during outpatient follow-up.

Controls. Children with severe asthma (not admitted to the PICU) were selected as controls from the PANDA study (NL67105.018.18) (*n* = 27). The PANDA study included children between 6 and 17 years old with asthma treatment step 3 or higher, according to the GINA guidelines [24]. For this study, only breath samples from patients with stable asthma (defined according to the [childhood] Asthma Control Test (ACT) > 19 points) were included in the analysis.

### 2.4. Exhaled Breath Collection for Metabolic Profiling

Exhaled breath was collected as previously described and according to ERS methodological standards [25]. In brief, patients were requested to breathe through an age-appropriate facemask attached to a carbon (Honeywell N) 6575001L and viral–bacterial filter (Bact-Trap™ Pharma Systems AB, Lysekil, Sweden) for ten tidal volumes to remove ambient VOCs through the filters. A T-piece connected both filters, which contained a one-way valve, securing air passing through the filter only during inhalation [26]. After the tenth exhalation, a maximal inspiration was followed by a short respiratory hold, during which a Mylar sampling bag was connected to the set-up. The patient exhaled one vital capacity in the complete set-up, inflating the sampling bag. An air sampling pump (GSP-300FT-2, GASTEC, Kanagawa, Japan) transferred the air of the sampled breath into a Tenax (Tenax GR SS 6 mm × 7”) sorbent tube (flow rate: 250 mL/min) and subsequently, the Tenax tubes were stored at 4 °C to ensure the stability of the captured VOCs for a maximum period of three months [27]. Due to ethical reasons, the samples from the cases were collected within 12 h after ending IV bronchodilators. Additionally, to ensure the most homogeneous effect possible of salbutamol in all samples, the sample was taken as long as possible after the last nebulisation, which differs between the patients but is always at least one hour. The half-life of nebulised salbutamol is estimated to be between 2.7 and 5 h [28].

### 2.5. VOC Analysis by Thermal Desorption Gas Chromatograph–Mass Spectrometry

The exhaled breath samples, stored in the sorbent tubes, were desorbed using the Markes TD100 autosampler and desorber (Cincinnati, OH, USA) and analysed by means of GC-MS, as described previously [19]. The sorbent tubes were heated to 250 °C and the VOCs captured on a cold trap at 25 °C before being rapidly injected onto an Inertcap 5MS/Sil GC column (30 m, ID 0.25 mm, film thickness 1 µm, 1,4-bis(dimethylsiloxy)phenylene dimethyl polysiloxane (Restek, Breda, The Netherlands)) with a flow of 1.2 mL/min. The oven temperature was kept isothermal at 40 °C for 5 min, then increased to 280 °C at 10 °C/min and kept isothermal at 280 °C for 5 min. VOCs were ionised using electronic ionisation (70 eV) and fragment ions detected using a quadrupole mass spectrometer (GCMS-QP2010, Shimadzu, Den Bosch, The Netherlands) with a scan range of 37–300 Da.

### 2.6. Data Analysis

Targeted VOCs were identified with the help of the Automatic Mass Spectral Deconvolution and Identification System (AMDIS, version 2.73), using biochemical reference data from the National Institute of Standards and Technology Mass Spectral Library (MS MIST Library, version 2.3).

For the quantitative analysis of the targeted VOCs, the peak area of the compounds was used. Abundance was considered as a derivate of this peak area, which is the integration of peak height in the chromatogram over time and is proportional to the amount of that compound present in the sample. For this analysis, raw peak areas in both groups were compared without using an internal standard. When a targeted compound was not identified in a sample, zero was assigned to its peak area to have numeric data for all targeted compounds in a sample.

The AMDIS and the MS NIST library were used for translating and analysing the data obtained by the TD-GC-MS. The raw GC-MS files were converted into netCDF files and analysed using AMDIS. AMDIS set out all RTs and *m*/*z* values represented in the samples. The MS NIST Library was consulted as a reference database for these RT and *m*/*z* values to identify the putative compound.

The default setting in AMDIS was compared to a manual setting used in previous research in a reduced dataset to find the most reliable setting (manual setting: minimum match factor: 60, compound width: 32, adjacent peak subtraction: one, resolution: high, sensitivity: very high, shape requirements: low. Type of analysis: simple). For both settings, a NET score was assigned for every identified compound, meaning the final match quality value for the match between the deconvoluted compound (in this case, the targeted compound) and the library spectrum, where its maximum value of 100 represents a perfect match. The manual setting was used for this study because of higher NET scores.

### 2.7. Statistical Analysis

To determine whether there was a significant difference between the presence of the targeted VOCs between cases (SAA) and controls (severe controlled asthma), a Fisher’s Exact Test was used, combined with a 95% confidence interval (CI). The data were not normally distributed, proven by the Shapiro–Wilk Test; therefore, the non-parametric Wilcoxon rank-sum test (also known as the Mann–Whitney U Test) was applied to compare the abundances between groups. Assuming that the VOCs associated with asthma exacerbations have higher abundances, the null hypothesis was set as ‘’equal’’ and the alternative was ‘’greater’’ for both tests. A significance level of *p* < 0.05 was determined to reject the null hypothesis. When a targeted compound was not identified in a sample, zero was assigned to the peak area. However, there is a possibility that multiple zeroes in the data could influence the reliability of the results. To confirm the significance of the results, the Wilcoxon rank-sum test was additionally performed for every targeted compound, excluding the samples where the peak area was zero (or not present). Statistical analyses were performed in R (version 4.2.1) through the RStudio interface.

### 2.8. Longitudinal Comparison of PICU Cases

To measure any effect of the asthma exacerbation or a viral infection, a common cause of an exacerbation, differences in breath profiles were assessed in cases between admission at the PICU and clinical recovery (approximately six weeks after discharge from the PICU). The ACT scores at admission and then follow-up were assessed for normality, after which a paired t-test was used to compare these scores. The compounds showed high grades of multicollinearity, as measured by the variance inflation factor (VIF). For this reason, the dimensionality of the data was reduced using principal component analysis (PCA) [29]. The PCs resulting from the admission samples were regarded as the training set, and the PCs of the revisit (clinical recovery) were calculated using the loading factors of the training set. Normal distribution of the resulting PCs was assessed using histograms and Q-Q plots. For comparison, the Wilcoxon signed-rank test was used.

## 3. Results

### 3.1. Systematic Review

The systematic review resulted in 914 eligible articles. Unfortunately, almost all articles described VOCs measured during stable asthma or used a different measuring technique. Hence, title and abstract and full text screening resulted in two articles [17,18]. Two more articles were included by the snowballing method [30,31]. Additionally, our research group recently identified seven differentiating VOCs present during an asthma exacerbation, which were included as well [19]. In total, this resulted in 29 VOCs reported by these studies, of which 25 VOCs were included in this paper (Table 1). Two VOCs were excluded because they were reported as “unknown”, and one VOC (1.2-methyl-4H-1,3-benzoxathiine) was not in the MS NIST library. With no reference data, targeting is not possible. Acetone, reported by Khamas et al., was excluded as well because it is seen as the confirmation VOC of a human sample representing lipolysis [32].

### 3.2. Study Participants

**Figure 1:**
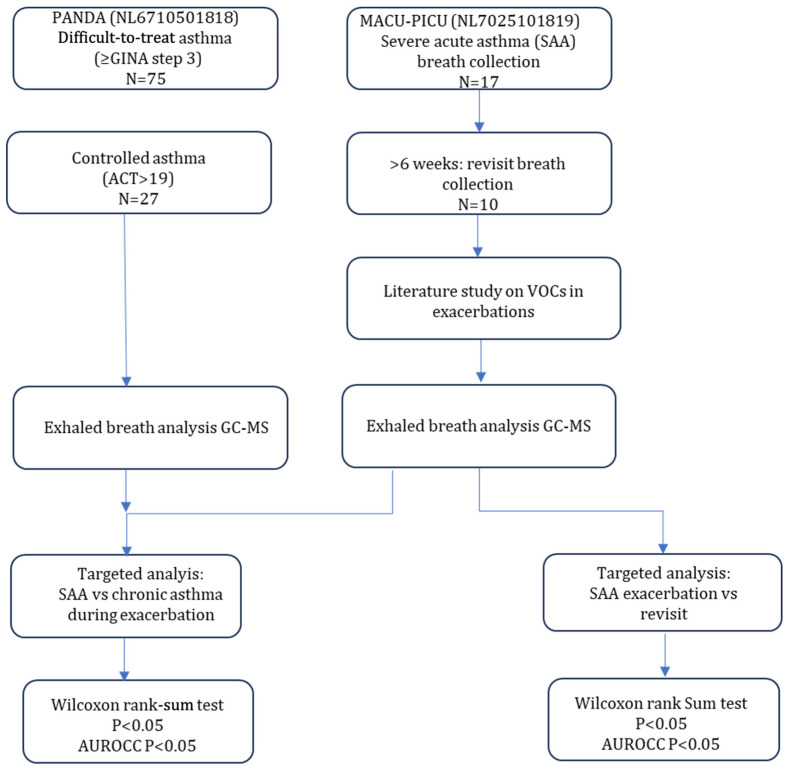
Schematic overview of patient inclusions. GINA: Global Initiative for Asthma; ACT: asthma control test; GC-MS: gas chromatography–mass spectrometry; AUROCC: area under the ROC curve.

A total of 17 eligible cases diagnosed with SAA were included in this study, of which 10 also had their breath samples taken at follow-up. Breath samples of 27 controls were used, resulting in a total of 44 breath samples (Figure 1). For a summary of the baseline characteristics, see Table 2.

History of asthma: diagnosed by physician; asthma admission: hospital admission in 12 months prior to study inclusion; PICU admission: admitted to a PICU for severe acute asthma; GINA treatment: prescribed treatment based on GINA criteria [24], where a higher number indicates a more advanced level of treatment regimen; family history of asthma: either parents or siblings; smoke exposure: smokers within household or exposure to cigarette smoke every day.

### 3.3. Targeted VOC Analysis in Cases vs. Controls

In total, 44 chromatograms were available for analysis. AMDIS identified a total of 655 compounds (SAA group, *n* = 297; control group, *n* = 358) in the breath samples. After verifying these putative compounds, 197/297 (66%) compounds in the SAA group and 216/358 (60%) were assumed to be correctly identified. The other 242 VOCs were excluded because of the high probability of misidentification. The percentage of incorrectly identified compounds was 34% and 40% in the SAA group and the controlled asthma group, respectively.

Sixteen of the 25 targeted VOCs were identified and were present in both groups (Table 3). Thirteen VOCs were more prevalent in cases compared to the controls, of which four were significant: methylethyl-1-benzene 59% vs. 15% (OR 7.75, 95% CI [1.99, ∞], *p* < 0.01); styrene 76% vs. 37% (OR 8.37, 95% CI [2.40, ∞], *p* < 0.05); ethylbenzene 82% vs. 52% (OR 4.19, 95% CI [1.06, ∞]); and benzaldehyde 82% vs. 52% (OR 4.19, 95% CI [1.06, ∞]). Contradictively, three VOCs were more prevalent in the controls: acetonitrile, benzene, and cyclohexane.

The concentration of the 16 targeted VOCs was compared in both groups, of which nine VOCs had a significantly higher concentration in the SAA group (nonanal, methylethyl-1-benzene, styrene, 2-ethyl-4-methylpentan-1-ol, octanal, p-xylene, ethylbenzene, benzaldehyde, and 2-octen-1-ol) compared to the compound area in the controlled asthma group. After excluding all samples without the targeted VOC and analysing the difference in peak areas between the groups in the remaining samples, five VOCs remained significantly higher in the SAA group compared to the controlled asthma group (methylethyl-1-benzene, 2-ethyl-4-methylpentan-1-ol, octanal, p-xylene, and benzene). Additionally, the area for 3-methylpentane was significantly less in the SAA group compared to the controlled asthma group (Table 4).

### 3.4. Longitudinal Analysis of Metabolic Breath Profiles of Cases

A total of 10 children were able to perform a breath analysis at follow-up. The mean ACT score of the children at admission was 15.3 (SD 6.16) compared to a mean ACT score at follow-up of 20.90 (SD 4.09). According to the Wilcoxon signed-rank test, these scores differed significantly (*p* = 0.03), indicating better asthma control at the revisit. In the breath samples of these ten patients, 16 VOCs were identified at admission. These 16 VOCs were pooled in a PCA, which transformed the 16 variables into six PCs with an eigenvalue of 1 or above, according to Kaiser criteria (Supplementary Table S1). These six PCs explained 91.5% of the total data variance. After the Wilcoxon signed-rank test, no PCs showed significant differences at the revisit compared to the admission samples.

## 4. Discussion

To our knowledge, this is the first study to use a targeted TD-GC-MS-based VOC analysis to profile the exhaled breath of children with severe asthma admitted to the PICU. In total, 16 VOCs previously linked to asthma attacks were identified in children with severe acute asthma. Four VOCs were significantly more often present in SAA (methylethyl-1-benzene, styrene, ethylbenzene, and benzaldehyde), while nine VOCs were detected in higher quantity in the SAA patients compared to the controlled severe asthma group (nonanal, methylethyl-1-benzene, styrene, 2-ethyl-4-methylpentan-1-ol, octanal, p-xylene, ethylbenzene, benzaldehyde, and 2-octen-1-ol). No significant differences were found in the breath profiles of cases during admission and revisit, indicating that these VOCs are not only present during a severe asthma attack but maintain over time and, therefore, could indicate a different disease subtype of severe acute asthma; however, as there is sparse research on endotypes in SAA comparing these to chronic asthma or exacerbations, more research is needed and currently being performed to further strengthen this indication.

For this study, we have applied a targeted approach, which has been used for VOCs before and appears to be able to discriminate between groups with clinically adequate sensitivity values [20]. With such a targeted approach, several VOCs were found in the breath profiles of children with SAA in this study. The identified VOCs can be related to several environmental processes, and these external factors are known to have an effect on (severe) asthma. For example, octanal, cyclohexane, and nonanal have been associated with oxidant-induced damage to unsaturated fatty acids in cell membranes [17,33]. Children with severe asthma are known to have increased biomarkers of oxidant stress because of persistent airway inflammation [34]. However, an in vitro study showed that in A549 cells undergoing oxidative stress, octanal was decreased and cyclohexane and nonanal were not significantly increased, making the link of these VOCs to oxidative stress questionable [35,36]. Cyclohexane, benzene, p-xylene, and ethylbenzene are related to solvent emissions [37]. Industrial process-related VOCs are cyclohexane, styrene, benzene, and p-xylene [38]. Children living close to roads with busy traffic or heavy trucks are more prone to develop asthma and are at a higher risk of hospital admission [39]. Cyclohexane, benzene, styrene, p-xylene, hexanal, and ethylbenzene reduce DNA methylation and cause airway remodelling [40]. Nonanal and hexanal are breakdown products from oleic acid, palmitoleic acid, oxidized linoleic acid and arachidonic acid [33]. Benzene, styrene, p-xylene, and ethylbenzene are present in tobacco smoke [33], and benzene, p-xylene, and ethylbenzene are also associated with vehicle exhaust, gasoline, and petroleum [41,42,43]. There is a large effect of (second-hand) smoke exposure on asthma and this is seen as an important risk factor for PICU admission for SAA [44]. Lastly, p-xylene is a critical precursor of ground-level ozone and secondary organic aerosol formation [45].

One of the main strengths of this study is the novelty of its prospective longitudinal design involving paediatric SAA patients studying metabolomics. The targeted compounds in this study were all substantiated by the available evidence in the literature, and breath measurements were performed in predefined sampling methods, thereby minimising environmental influences. Furthermore, GC-MS analysis, which we used to analyse our breath samples, is considered the gold standard for exhaled metabolite studies, and we employed supported compound identification methods to prevent false VOC discovery. Additionally, we found the same VOCs in the admission and follow-up samples for the cases, which confirms their presence in the breath of the target population.

However, limitations are present. Although the majority of the samples were taken in a standardised manner, two patients consumed food <2 h prior to sampling, and three patients underwent recent nebulisation or were still on a salbutamol IV at 0.5 mcg/kg/min when sampling. The literature is ambiguous about the influence of diet, either stating that food is most dominant after one hour of ingestion or stating that the patient should not eat one hour prior to sampling. The influence of salbutamol exposure on the externally produced VOCs is expected to be small due to the low occurrence and the targeted analysis performed.

Furthermore, the possible effects of oxygen therapy at the time of sampling are especially important at the PICU. In our study, two patients still received oxygen at the time of sampling. The air entering the lungs via the oxygen system may contain VOCs produced by the system itself and bypass the VOC carbon filter of the setup. The exogenous VOCs produced by the oxygen system and the endogenous effect of oxygen therapy on VOCs are currently unknown. Another factor that could generate variability in exhaled breath samples is the circadian rhythm [46]. Due to the small timeframe in which the sampling could be performed at the PICU, this was not considered for the current study.

Also, it was found that nine targeted VOCs were found in significantly higher amounts in SAA. The peak area, however, is sensitive to internal and external conditions and is dependent on the most dominant VOCs in the sample. Normally, an internal standard is added, the concentration of which is known. The areas of the targeted compounds can then be compared to the peaks of the internal standard, and the concentration can be estimated more accurately. Normalisation of the data based on the highest VOCs is another option to overcome this limitation. Unfortunately, this was beyond our possibilities, and raw peak areas in both groups were compared. For future studies, it would be valuable to repeat this study and apply those measures.

Finally, different storage methods should be considered. For this study, the Tenax tubes were stored in a refrigerator at 4 °C for a duration of three months maximum before they were injected into the GC-MS. All samples were preferred to be analysed in the same run to minimise external influences. However, Kang et al. investigated the degree of deterioration of breath samples and concluded that storage at 4 °C should not exceed 30 days and advised storing at −80 °C for better preservation of the VOCs [47]. To see if the deterioration of VOCs could be the case in our samples, we manually compared the presence of VOCs in the breath samples preserved for the longest period (three months) and the shortest period (four days). Deterioration appeared to be unlikely, as 11 versus nine targeted VOCs were identified in the oldest and the newest samples, respectively.

In the future, further validation of the above-mentioned VOCs in a larger patient group with SAA at the PICU will be performed. Additionally, other metabolomic tests, such as microbiomics, viromics, and transcriptomics, will be collected and integrated with breathomics to explore possible associations. The main goal is to improve early diagnosis and monitoring of SAA patients by better understanding SAA’s molecular pathophysiology, thereby facilitating a timely adjustment of medication. Moreover, prediction of the therapeutic response could optimise the efficiency of current therapies and support the implementation of emerging novel targeted therapies such as biologicals.

## 5. Conclusions

This study presents the first step towards metabolic profiling and extensive phenotyping of paediatric SAA patients using TD-GC-MS-based VOC analysis profiles in exhaled breath. Several VOCs that are linked to an asthma exacerbation were more prevalent or identified in higher concentrations in the breath of SAA patients compared to controlled asthma patients. Therefore, breath metabolomics may enable early-stage recognition of severe asthma attacks and may lead to more prompt interventions in children with severe acute asthma. This article addresses the pressing clinical need to improve treatment for this patient population and further personalise therapy regimens.

## Figures and Tables

**Table 1:** Summary of the included studies and their reported VOCs associated with uncontrolled asthma.

Included VOCs Reported by Study	Study Population (Number of Patients)	General Aim	Main Results
Van Vliet et al. [17] 1, 2-dimethylcyclohexane 2-ethylhexanal Octanal 6, 10-dimethyl-5, 9-undecadien-2-one Nonanal 2-methylfuran 3-methylfuran	Children with asthma (96)	Discriminate children with persistently controlled and uncontrolled asthma and predict an exacerbation	Sensitivity and specificity of predicting an asthma exacerbation 14 days after sampling were 88% and 75% based on 7 VOCs
Shahbazi Khamas, S et al. [19] 2,4,4-trimethyl pentene 1-phenylethanone Benzaldehyde Styrene methyl-ethyl-1-benzene Ethylbenzene	Children with asthma (40)	Identify different VOCs between severe, uncontrolled asthma and controlled asthma	7 VOCs were able to discriminate between uncontrolled asthma AUC 0.812 95% CI [0.657–0.968]
Robroeks et al. [18] P-xylene 3-methylpentane 2-ethyl-4-methyltrimol but-1-enylbenzene 4,6,9-nonadecatriene 3-methylidenepent-1-ene Cyclohexane 2-octen-1-ol Benzene	Children with asthma	Prediction of asthma exacerbation	6 VOCs resulted in the most optimal model to predict exacerbation (correct classification 96%, sensitivity 79% and specificity 100%)
Brinkman et al. [30] Methanol Acetonitrile 4-methylbicyclo [2.2.2]octan-1-ol	Adolescents and adults with asthma (23)	To discriminate between clinical stable and unstable asthma	3 VOCs discriminated correctly between loss of control and controlled asthma with accuracies of 68% and 77%
Delfino et al. [31] Benzene	Children with asthma (21)	Environmental factors and their effect on asthma exacerbation	Non-significant association between benzene and severe asthma symptoms (OR 2.03, 95% CI, [0.80–5.11])

**Table 2:** Patient characteristics.

	Cases Severe Acute Asthma (n = 17)	Controls Controlled Severe Asthma (n = 27)
Age (mean)	2–17 (9.3)	6–17 (12.2)
Sex, male (%)	8 (47.1%)	21 (77.8%)
Ethnicity (%)		
Caucasian	9 (52.9%)	14 (51.8%)
History of asthma (%)		
Yes	12 (70.6%)	27 (100%)
Asthma admission (%)		
Yes	7 (41.2%)	15 (55.6%)
Previous PICU admission (%)		
Yes	3 (17.6%)	11 (40.7%)
Asthma treatment step (%)		
0	4 (23.5%)	0 (0%)
1	6 (35.3%)	0 (0%)
2	0 (0%)	0 (0%)
3	1 (5.9%)	2 (7.4%)
4	3 (17.6%)	5 (18.5%)
5	3 (17.6%)	20 (74.1%)
Atopy (%)		
Yes	12 (70.6%)	20 (74.1%)
No	5 (29.4%)	7 (25.9%)
Family history of asthma (%)		
Yes	11 (64.7%)	14 (51.9%)
No	6 (35.3%)	13 (48.1%)
Family history of atopy (%)		
Yes	15 (88.2%)	23 (85.2%)
No	2 (11.8%)	4 (14.8%)
Smoke exposure at home (self-reported) (%)		
Yes	3 (17.6%)	4 (14.8%)
No	14 (82.4%)	23 (85.2%)

**Table 3:** Targeted analysis of VOCs associated with asthma exacerbation with adopted RT in SAA and CA.

Targeted VOCs	Adopted RT (min)	SAA (n = 17) (%)	CA (n = 27) (%)	Odds Ratio (95% CI)	*p* Value
Nonanal	17.30	17 (100%)	24 (89%)	∞ [0.61, ∞]	*p* = 0.14
Methylethyl-1-benzene	13.62	10 (59%)	4 (15%)	7.75 [1.63, 45.5]	*p* = 0.01
Styrene	12.81	13 (76%)	10 (37%)	5.30 [1.21, 28.8]	*p* = 0.02
2-ethyl-4-methylpentan-1-ol	15.76	17 (100%)	25 (93%)	∞ [0.12, ∞]	*p* = 0.51
Octanal	15.23	16 (94%)	23 (85%)	2.73 [0.24, 145.35]	*p* = 0.63
*p*-xylene	12.87	8 (47%)	6 (22%)	3.0, 14.23]	*p* = 0.11
Ethylbenzene	12.19	14 (82%)	14 (52%)	4.19 [0.87, 27.96]	*p* = 0.06
Benzaldehyde	14.48	14 (82%)	14 (52%)	4.19 [0.87, 27.96]	*p* = 0.06
2-octen-1-ol	18.81	8 (47%)	6 (22%)	3.03 [0.69, 14.23]	*p* = 0.11
2,4,4-trimethyl pentene	8.88	11 (65%)	12 (44%)	2.25 [0.56, 9.81]	*p* = 0.23
3-Methylpentane	8.25	4 (24%)	3 (11%)	2.41 [0.35, 19.05]	*p* = 0.41
1-Phenylethanone	16.77	4 (24%)	4 (15%)	1.74 [0.28, 11.13]	*p* = 0.69
2-Ethylhexanal	14.22	11 (65%)	14 (52%)	1.68 [0.42, 7.29]	*p*= 0.54
Acetonitrile	3.57	4 (24%)	7 (26%)	0.88 [0.16, 4.35]	*p* = 1.0
Benzene	5.97	2 (12%)	6 (22%)	0.47 [0.04, 3.15]	*p* = 0.46
Cyclohexane	3.72	13 (76%)	22 (81%)	0.74 [0.13, 4.46]	*p* = 0.72
1, 2-dimethylcyclohexane	-	-	-	-	-
but-1-enylbenzene	-	-	-	-	-
3-methylidenepent-1-ene	-	-	-	-	-
2-methylfuran	-	-	-	-	-
3-methylfuran	-	-	-	-	-
4,6,9-nonadecatriene	-	-	-	-	-
6, 10-dimethyl-5, 9-undecadien-2-one	-	-	-	-	-
4-methylbicyclo[2.2.2]octan-1-ol	-	-	-	-	-
Methanol	-	-	-	-	-

**Table 4:** Quantitative targeted analysis of the peak area of VOCs associated with asthma exacerbation in SAA and CA.

Targeted VOCs	Median Peak Area SAA (×10^6^) [1–3 IQR]	Median Peak Area CA (×10^6^) [1–3 IQR]	*p* Value	Median Peak Area SAA (×10^6^) [1–3 IQR] *	Median Peak Area CA (×10^6^) [1–3 IQR] *	*p* Value *
Nonanal	60 [32–87]	18 [9.7–29]	*p* = 0.001	60 [32–87]	14 [6.7–29]	*p* = 0.00
Methylethyl-1-benzene	2.3 [1.5–3.7]	12 [7.6–16]	*p* = 0.84	1.4 [0–2.6]	0 [0–0]	*p* = 0.00
Styrene	5.5 [2.4–31]	5.7 [1.7–31]	*p* = 0.78	2.5 [1.2–12]	0 [0–2.4]	*p* = 0.02
2-Ethyl-4-methylpentan-1-ol	12 × 10^1^ [89–19 × 10^1^]	42 [35–80]	*p* = 9.6 × 10^−6^	12 × 10^1^ [89–19 × 10^1^]	42 [35–79]	*p* = 0.00
Octanal	24 [12–31]	12 [6.4–16]	*p* = 0.03	23 [11–31]	12 [4.3–18]	*p* = 0.02
*p*-xylene	4.6 [3.4–7.6]	4.8 [1.9–12]	*p* = 0.03	0 [0–4.2]	0 [0–0]	*p* = 0.04
Ethylbenzene	6.8 [2.7–13]	3.2 [1.4–11]	*p* = 0.60	2.9 [1.8–9.5]	0.49 [0–5]	*p* = 0.04
Benzaldehyde	2.9 [1.4–20]	1.4 [0.8–13]	*p* = 0.70	1.7 [0.6–10]	0.21 [0–6.6]	*p* = 0.05
2-octen-1-ol	16 [12–18]	11 [2.9–24]	*p* = 0.95	0 [0–16]	0 [0–0]	*p* = 0.09
2,4,4-trimethyl pentene	8.4 [5.7–20]	8.5 [7.1–11]	*p* = 0.53	5.3 [0–8.5]	0 [0–7.5]	*p* = 0.15
3-Methylpentane	0.50 [0.15–0.88]	2.6 [1.5–3.7]	*p* = 0.06	0 [0–0]	0 [0–0]	*p* = 0.41
1-phenylethanone	3.5 [3.3–3.7]	5.4 [4.0–7.3]	*p* = 1.0	0 [0–0]	0 [0–0]	*p* = 0.48
2-Ethylhexanal	3.3 [2.2–4.7]	4.4 [2.1–9.8]	*p* = 0.69	2.1 [0–3.8]	0.97 [0–3.6]	*p* = 0.59
Acetonitrile	0.37 [0.072–2.8]	0.23 [0.21–2.3]	*p* = 0.93	0 [0–0]	0 [0–0.016]	*p* = 0.90
Benzene	4.2 [2.9–5.5]	0.81 [0.73–0.99]	*p* = 0.07	0 [0–0]	0 [0–0]	*p* = 0.53
Cyclohexane	14 [12–16]	16 [6.2–24]	*p* = 0.53	13 [7.0–16]	15 [1.8–23]	*p* = 0.47
1, 2-dimethylcyclohexane	-	-	**-**	-	-	-
but-1-enylbenzene	-	-	**-**	-	-	-
3-methylidenepent-1-ene	-	-	**-**	-	-	-
2-methylfuran	-	-	**-**	-	-	-
3-methylfuran	-	-	**-**	-	-	-
4,6,9-nonadecatriene	-	-	**-**	-	-	-
6, 10-dimethyl-5, 9- undecadien-2-one	-	-	**-**	-	-	-
4-methylbicyclo[2.2.2]octan-1-ol	-	-	**-**	-	-	-
Methanol	-	-	**-**	-	-	-

* Distribution in all patients in the cohort, where a value of zero is allocated to all samples without the targeted VOC identified.

## Data Availability

The data that support the findings of this study are available upon reasonable request. Please contact the corresponding author, Dr. Berber Kapitein, for further information on b.kapitein@amsterdamumc.nl.

## Supplementary Materials

The following supporting information can be downloaded at https://www.mdpi.com/article/10.3390/metabo15040247/s1, Table S1: Rotated factor loading for each VOC after PCA for longitudinal analysis.

## Abbreviations

The following abbreviations are used in this manuscript:

SAA	Severe Acute Asthma
PICU	Paediatric Intensive Care Unit
TD-GC-MS	Thermal Desorption Gas Chromatography–Mass Spectrometry
VOC	Volatile Organic Compound
COPD	Chronic Obstructive Pulmonary Disease
MACU-PICU	Multisystem phenotyping in severe acute asthma at the PICU
PANDA	Paediatric Asthma Non-Invasive Diagnostic Approaches
IV	Intravenously
GINA	Global Initiative for Asthma
ACT	Asthma Control Test
ERS	European Respiratory Society
AMDIS	Automatic Mass Spectral Deconvolution and Identification System
MS-NIST	National Institute of Standards and Technology Mass Spectral Library
RT	Retention Time
PC	Principal Components
