# Supplementary material for: Uncovering Non-Invasive Biomarkers in Paediatric Severe Acute Asthma Using Targeted Exhaled Breath Analysis
Source: Metabolites. 2025 Apr 3;15(4):247. doi: 10.3390/metabo15040247 (PMC12029713; doi:10.3390/metabo15040247)
Supplement: Supplementary file 1 [file metabolites-15-00247-s001.zip › metabolites-3528637-supplementary.pdf]

metabolites

MDPI

Supplemental Materials

Supplement Table 1. Rotated factor loading for each VOC after PCA for longitudinal analysis.

PC	Rotated factor loading	VOC	% of Variance	Cumulative %
1	0.965	Styrene		
	0.930	Benzene (1-methylethyl)-	25.12%	25.12%
2	0.924	Octanal		
	-0.818	Benzene		
	0.666	Nonanal	20.05%	45.17%
3	0.921	Pxylene		
	0.724	1-pentanol, 2-ethyl		
	0.705	Cyclohexane	15.65%	60.82%
4	0.896	Acetophenone		
	0.771	2-octen-1-ol	12.48%	73.30%
5	0.846	Ethylbenzene		
	-0.699	Hexanal 2-ethyl		
	0.663	Benzaldehyde		
	0.583	Pentane 3-methyl	10.56%	83.85%
6	0.947	2,4,4 trimethyl		
	0.634	Acetonitrile	7.60%	91.45%

Rotated factor loadings defined as numbers that represent the contribution of a variable to a factor in principal component analysis. Loadings closer to 1 or -1 indicate a higher contribution. Positive factor loadings refer to a positive relationship between the compound and the PC, a negative factor loading refers to a negative relationship.
